# New York Medical Intelligencer

**Published:** 1845-10

**Authors:** 


NEW YORK MEDICAL INTELLIGENCER.

This is a new aspirant for professional favour. The first number, which is before us, consists of sixteen quarto pages, double columns, and closely printed. The Editor, D. S. Meikleham, M. D., in a neatly written prospectus, announces the object of the publication to be altogether eclectic—to collect whatever is valuable in the European Medical Journals, and present it to his readers without the admixture of the local and uninteresting matter which constitutes so large a portion of their contents; imitating, in that respect, the "Periscopes" of some of our existing Journals, and the "Record" of our own. It is designed to be issued monthly, at two dollars a year, payable in advance.

